# Exploring the potential of ume-derived proanthocyanidins: novel applications for blueberry preservation

**DOI:** 10.3389/fmicb.2023.1265993

**Published:** 2023-09-27

**Authors:** Lishan Liang, Honghao Qiu, Yuntong Liu, Yingjie Liu, Luo Weng, Wenting Zhong, Fanxin Meng

**Affiliations:** ^1^College of Pharmacy and Food Science, Zhuhai College of Science and Technology, Zhuhai, China; ^2^College of Life Science, Jilin University, Changchun, China

**Keywords:** ume, proanthocyanidins, preservative, antibacterial, preservation of freshness

## Abstract

Proanthocyanidins (PCs) extracted from ume have many well-known functional properties. The aim of this study was to explore a novel natural food preservative using ume plum pulp proanthocyanidins (UPPP). The crude product of PCs from ume plum was obtained by using ethanol as extraction solvent and ultrasonic-assisted extraction, and then the pure product of UPPP was obtained by purification with AB-8 resin. The bacteriostatic ability of UPPP and the freshness preservation effect on blueberry were analyzed. The results showed that UPPP had a high inhibitory effect on *Staphylococcus aureus* (MIC of 1.563 mg/mL) and *Escherichia coli* (MIC of 3.125 mg/mL). Findings revealed that, in comparison to 0.02% potassium sorbate, blueberries treated with a high concentration of UPPP in a dipping treatment displayed superior quality maintenance after 7 days of storage at 4°C. Importantly, analysis of the various metrics showed that treatment with UPPP was significantly better compared to blueberries treated with 0.02% potassium sorbate. For example, the decay rate, weight loss, and total number of colonies of blueberries treated with 0.02% potassium sorbate were 55.56, 3.48%, and 4.24 ± 0.07 log CFU/mL, whereas the values of the above indexes for blueberries treated with 25 mg/mL of UPPP were 22.22, 3.09%, and 3.10 ± 0.17 log CFU/mL, respectively. Conversely, blueberries that were not dipped in any preservative displayed signs of deterioration as early as the 3rd day of the storage period, highlighting the potential of UPPP as a valuable method for preserving fruits and vegetables. Therefore, UPPP holds great promise as an innovative natural food preservative, effectively enhancing food safety, quality, and extending shelf-life.

## 1. Introduction

Ume is processed from the dried nearly ripe fruit of *Prunus mume (Sieb.) Sieb. et Zucc.* in the Rosaceae family. As a dual-use species of medicine and food, ume has received widespread attention from both China and foreign countries, and can be eaten raw or used to make preserved fruit, jam or sour plum soup, which contains a large number of organic acids, flavonoids, lipids and other components (Shang and Bai, 2022). Ume has the efficacy of astringing the lung, astringent intestines, generating fluids, and tranquilizing roundworms, and is commonly used in the treatment of lung deficiency and prolonged cough, prolonged diarrhea and dysentery, deficiency heat and thirst, roundworm convulsions, vomiting, and abdominal pain (Wen et al., 2023). Modern pharmacological studies have shown that ume, as a natural health care product and functional food, has antibacterial, antitumor, antioxidant, hypoglycemic, and inhibit kidney stones (Zhang et al., 2016, 2017; Gong et al., 2021).

PCs are a class of polyphenolic compounds widely found in nature, condensed from different amounts of catechins and epicatechins, with good antioxidant properties and free radical scavenging ability (Zhao et al., 2010; Lindsey et al., 2018). Thirteen PCs components, including two monomers of catechin and epicatechin, four B-type dimers, three A-type dimers, two B-type trimers, one A-type trimer, and one B-type tetramer, were present in ume (Horinishi et al., 2021). Currently PCs are widely used as nutritional fortifiers, natural antioxidants, natural preservatives, etc. in food, pharmaceuticals, cosmetics and other fields (Lv et al., 2020). PCs exhibited high antibacterial activity on many different pathogenic bacteria. Karioti et al. showed that procyanidin B_3_ and prodelphinidin C exhibited the minimum inhibitory concentration (MIC) and minimum bactericidal concentration (MBC) on *E. coli* in the comparison with the other 12 phenolics from *Quercus ilex* leaves (Karioti et al., 2011). Many clinical trials have shown that cranberry juice and its derivatives are useful mainly because of the anti-adhesive properties of Cranberry PCs, which have an important effect in the prophylaxis of recurrent urinary tract infection (UTI; Micali et al., 2014). Antibiofilm properties of Cranberry PCs on *Pseudomonas aeruginosa* were also observed (Ulrey et al., 2014), indicating that PCs might be a useful therapy against biofilm-mediated infections caused by *P. aeruginosa*. In addition, PCs also play an important role in fruit preservation. It was found that different concentrations of PC mulching treatment had preservation effects on peach, which could reduce the fruit water loss rate, delay sugar accumulation, delay the appearance of respiratory peak time, and reduce the peak value of respiratory peak to different degrees (Liu et al., 2019). The PCs of *Sorbus Melanocarpa* could effectively reduce the MDA content and inhibit the bacterial growth of apples, which obviously reflected its freshness preservation effect (Zhang, 2021).

Fresh fruit is not only delicious, but also packed with essential nutrients like vitamins, polyphenols, dietary fiber, and minerals that are crucial for our daily diets (Zhang and Jiang, 2019). Research has shown that consuming fresh fruit can help prevent various diseases, including stomach cancer, colon cancer, heart disease, and diabetes (Lunet et al., 2005; Clifton et al., 2014). Blueberries are rich in anthocyanins, vitamins, folic acid, dietary fiber, minerals and other nutrients and functional components (Patel, 2014), which have the effect of improving human immunity, improving eyesight, anti-aging, etc., and the blueberry pulp is delicate and juicy, with a suitable sweet and sour taste, which is loved by consumers. Blueberries are widely marketed in fresh, frozen and processed forms for different food uses (Cantín et al., 2012). Therefore, effective storage of blueberries plays an important role. The preservation of blueberries is crucial from an economic standpoint to ensure their long-term supply and minimize potential economic losses. Due to the susceptibility of blueberries to microbial and enzymatic degradation, employing effective preservation methods can extend their shelf life. Furthermore, blueberries are a valuable source of antioxidants, vitamins, and minerals, making it important to preserve their quality and nutritional attributes for consumer health. Effective preservation methods aid in retaining the nutritional value of blueberries, allowing consumers to enjoy their benefits even after prolonged storage. Additionally, blueberries find wide applications in the baking, confectionery, and beverage industries. Maintaining the quality of blueberries is vital for these industries to consistently incorporate high-quality ingredients and produce products with desirable sensory attributes. Lastly, preserving blueberries helps reduce food waste. By extending the shelf life of blueberries, they can be stored and distributed effectively, minimizing spoilage and reducing the quantity of discarded products. However, postharvest blueberries are still active organisms that deteriorate rapidly as they age, leading to nutrient degradation. Furthermore, fresh fruits and vegetables are highly vulnerable to microbial infestation, with reports of *Escherichia coli*, *Salmonella*, *Staphylococcus aureus*, and *Listeria monocytogenes* growth on blueberries (Popa et al., 2007; Miller et al., 2013; Sun et al., 2014), resulting in significant postharvest losses. As a result, scientists have been researching effective preservation techniques such as 1-MCP and SO_2_, commonly utilized for blueberry preservation. However, improper application of 1-MCP can lead to abnormal ripening of climacteric fruits, negatively impacting fruits quality (Song et al., 2020). To address safety concerns associated with chemical preservative residues, finding natural fruit preservatives has become the primary objective.

PCs can be used as a natural preservative for fruit preservation due to its antioxidant and bacteriostatic activities. However, the utilization of PCs extracted from ume as natural food preservatives for fruit preservation has not been thoroughly examined. Based on the activity of PCs, UPPP was extracted and purified from ume plums, and it was hypothesized that UPPP could effectively inhibit the growth of common pathogenic bacteria associated with fruit spoilage, and the preservative efficacy of UPPP-treated blueberries was compared with that of blueberries treated with potassium sorbate, a traditional food preservative. To study the bacteriostatic ability of UPPP and its preservation effect on blueberries, various parameters including decay rate, weight loss, hardness, color change, soluble solids, peroxidase (POD) activity, superoxide dismutase (SOD) activity, and colony counts were determined in an attempt to unravel the potential of UPPP as a natural food preservative especially for blueberry preservation. This study was expected to provide valuable insights into the potential application of UPPP as natural food preservatives. The findings would contribute to enhancing food safety, quality, and shelf life of blueberries and aid in the development of safer and more effective preservation technologies. In addition, it was also conducive to the active development of new resources such as PCs, which would ultimately maximize the utilization of ume resources.

## 2. Materials and methods

### 2.1. Materials

Ume (purchased from Dachuan, Sichuan, China), sterile TTC solution (0.5%; Guangdong Huankai Biotechnology Co., Ltd.), *Staphylococcus aureus* (*S. aureus*; ATCC 29231, GenBank: U77328 *S. aureus* staphylokinase gene, partial cds.), *Escherichia coli* (*E. coli*; ATCC 25922, GenBank: AF038431 *E. coli* DNA gyrase A (gyrA) gene, partial cds.), *Aspergillus niger* (*A. niger*; ATCC 16888, GenBank: FJ195350 ITS including 5.8S rRNA gene; purchased from Guangdong Microbial Strain Conservation Center, China), POD kit, SOD kit (Beijing Solepol Technology Co., Ltd., China), Handy Plate® Aerobic Count Plate (HP001, Guangdong Huankai Biotechnology Co., Ltd.).

### 2.2. Extraction and purification of UPPP

The optimal extraction conditions of UPPP were obtained by single-factor and response surface experiments. An appropriate amount of degreased ume powder was weighed, sieved to 0.25 mm, and UPPP was extracted with an ultrasonic extractor (XH-300A, Xianghu Technology, China) with 49% ethanol in the ratio of 15:1 at the ultrasonic power of 155 W. The extraction time was set to 48 min, and the extraction temperature was 45°C. The crude extract was centrifuged for 10 min (8,000 rpm/min), the supernatant was transferred, and 1/4 of the supernatant volume was evaporated by rotation to obtain the concentrated liquid. The concentrated extract was diluted to a certain concentration and purified by AB-8 macroporous adsorption resin.

During the purification process, the pre-treated AB-8 macroporous resin was loaded into a 30 mm × 300 mm column. The pH value was adjusted to 4, and then the concentrated extract solution obtained above was diluted to a concentration of 1.0 mg/mL and loaded into the chromatographic column. The loading flow rate was 1.25 mL/min and the loading volume was 2 BV. After loading, the sample was allowed to stand for 4 h, followed by washing with distilled water until the effluent was colorless to remove soluble proteins, sugars, and pigments. Subsequently, elution was carried out using 60% ethanol eluent, with a flow rate of 1.25 mL/min and an elution volume of 4 BV. The eluate was collected and subjected to rotary evaporation and freeze-dried at −40°C using a vacuum freeze dryer (FD-250101, FTFDS, China) and stored away from light to obtain the purified UPPP (Hao et al., 2021).

**Figure 1:**
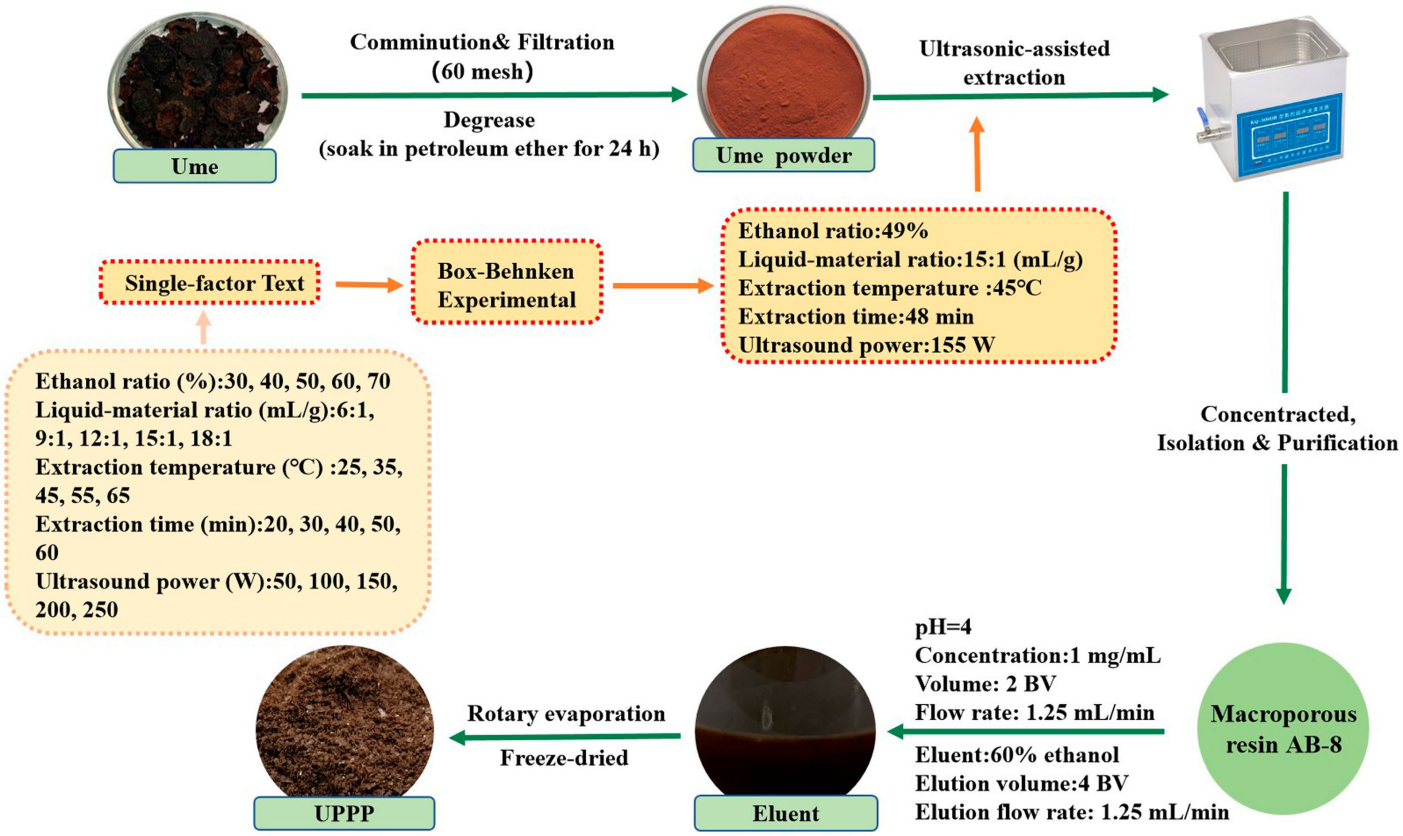
The extraction and purification procedure of UPPP.

The above experimental steps were repeated three times. The extraction, separation and purification process of UPPP is shown in Figure 1.

### 2.3. Content identification of UPPP

The PCs standard (Lot. Number: N12HB201033, UV ≥ 95%, Shanghai Yuanye Bio-Technology Co., Ltd., China) was prepared into concentration gradient solutions of 2 mg/mL, 3 mg/mL, 4 mg/mL, 5 mg/mL and 6 mg/mL, and the absorbance value was determined according to the vanillin hydrochloride method (Jiang et al., 2021), and 502 nm was selected. The value of absorbance was determined by using a microplate reader (Epoch, Bio-Tek Instruments inc., America). The extracted sample solution was diluted into an appropriate concentration gradient and determined with reference to the standard curve method, and then the PCs in the UPPP extract was rapidly determined according to the conversion of the standard curve. The experiment was repeated three times. The content of UPPP was calculated according to the following formula (Liu et al., 2022):


$$\mathrm{UPPP content}\;\left(\mathrm{mg}/g\right)=\frac{\mathrm{cvd}}{m}$$

c: concentration of UPPP (mg/mL); v: sample volume (mL); d: dilution multiple; m: mass of skimmed ume powder (g).

The purity of PCs in UPPP was calculated according to the following equation:


$$\mathrm{Purity}\left(\%\right)=\frac{\mathrm{mass of}\;\mathrm{PCs}\;\mathrm{in UPPP purification}}{\mathrm{mass of UPPP purification}}\times 100\%$$

### 2.4. Fourier transform infrared spectroscopy

Completely dried KBr, UPPP, and PCs standards were ground in a dry environment in proportion (2 mg sample/1,000 mg KBr), thoroughly mixed in an onyx mortar, pressed, and scanned with a FTIR spectrometer (Prestige-21, Shimadzu, Japan) in the range of 4,000 cm^−1^ to 400 cm^−1^ (Levdanskiy et al., 2021).

### 2.5. Determination of physicochemical properties of UPPP

To identify the presence of PCs in the purified products, the procedure described in the literature (Lawrence et al., 1985; Luo et al., 2020) was followed. The identification was done by employing a colorimetric reaction of UPPP with NH₄Fe(SO₄)₂ and vanillin under acidic conditions. In the reaction mechanism involving iron salt, the C-C bond in the PCs structure was broken, resulting in the formation of dark red anthocyanin ions. This reaction took place when heated under acidic conditions with iron salt acting as a catalyst. On the other hand, the reaction mechanism with vanillin occurred through the condensation of resorcinol or phloroglucinol, present in PCs, with the phenolic compound in vanillin, leading to the production of red substances. This reaction took place in acidic conditions. Both methods enabled the detection and quantification of the presence and concentration of PCs. In the experimental process, 1 mg of UPPP was dissolved in 15 mL of ethanol solution, followed by the addition of 6.0 mL of n-butanol-hydrochloric acid solution and 0.2 mL of 2% ammonium ferric sulfate solution. The color change of the UPPP solution was observed. Additionally, 1 mg of UPPP was dissolved in 15 mL of ethanol solution, and a few drops of 4% vanillin-hydrochloric acid solution were added, and the color change of the UPPP solution was observed. The experiment was repeated three times.

### 2.6. Antibacterial activity

#### 2.6.1. Measurement of bacterial inhibition zone

The bacteria preserved in the test tubes were transferred to 100 mL of sterile LB medium by a sterile inoculation ring, and placed in a 37°C, 170 r/min shaker for 24 h of constant temperature cultivation and then transferred three times for the activation of the strains (Yue et al., 2023), and the activated test strains were suspended in sterile saline solution until obtaining suspensions with density equal to 0.5 McFarland turbidity standard (10^8^ CFU mL^−1^) and, then, tenfold diluted twice (Lin et al., 2022).

The fungi was removed from −80°C for recovery, inoculated in PDA medium at 28°C for 5 days (Yang et al., 2022), and then the spores were washed down with sterile saline, until obtaining suspensions with density equal to 0.5 McFarland turbidity standard (10^8^ CFU mL^−1^) and, then, tenfold diluted twice (Lin et al., 2022).

The bacteriostatic activity of UPPP was evaluated against common bacteria (*S. aureus*, and *E. coli*) and fungi (*A. niger*) by using the agar well diffusion method (Manivasagan et al., 2013) with reference to the experimental method of Yu et al. with slight modification (Yu et al., 2018). 100 μL of bacteria at a concentration of 1 × 10^6^ CFU/mL was added to NA medium, and wells (6 mm in diameter) were punched in the medium, and then 90 μL of UPPP and sterile water (blank control) were added to the medium wells, respectively. 100 μL of fungal spore solution (concentration of 1 × 10^6^ CFU/mL) was added to PDA medium, and holes were punched in the medium (6 mm in diameter), then 90 μL of UPPP and sterile water (blank control) were added to the medium wells, respectively, and incubated at 37°C (24 h for bacteria and 72 h for fungi; Chen et al., 2022). The size of bacterial inhibition zone was measured and photographed. Materials and tools for each experiment were autoclaved and an ultra-clean bench was used for all operations. The above test steps were repeated three times.

#### 2.6.2. Minimum inhibitory concentration

The MIC of UPPP was measured by micro-broth dilution method by referring to the experimental method of Andressa Veiga et al. with slight modifications (Veiga et al., 2019). 100 μL of LB medium mixed with sterile TTC solution (0.5%) was added to a sterile 96-well plate, 100 μL UPPP with a concentration of 25 mg/mL was added to the first well, and then the mixture was diluted sequentially to the twelfth well by twofold dilution method, and finally 100 μL of bacterial solution (1 × 10^6^ CFU/mL) was added to each well, then the 96-well plates were incubated at 37°C in a thermostatic microbiological incubator for 24 h. The bacterial growth of each well was observed and analyzed, and the lowest concentration with no bacterial growth and no OD increase was defined as MIC value (Kowalska-Krochmal and Dudek-Wicher, 2021). The experiment was repeated three times and no UPPP was added as a control in the blank group.

The MIC of fungi was basically the same as the above steps, in which the LB medium was replaced with PDB medium, and the incubation temperature and time were 30°C and 5 days (Ali et al., 2022), respectively.

#### 2.6.3. Growth curve

Bacterial growth curve analysis was performed according to the Feng et al. (2022) method with modifications to determine the effect of UPPP on the growth curves of *S. aureus* and *E. coli* (Feng et al., 2022). The analysis was performed in sterile 96-well plates. The MIC values of UPPP on *S. aureus* and *E. coli* determined in previous experiments were used in this test. 200 μL of LB medium was added to a sterile 96-well plate, followed by UPPP at concentrations of 0.125 MIC, 0.25 MIC, 0.25 MIC, and 1 MIC and mixed thoroughly. LB medium without extracts was used as a blank control. Then, 20 μL of bacterial solution (1 × 10^6^ CFU/mL) was added to each well and incubated at 100 rpm/min in a thermostatic air-bath shaker (SKY-2112B, Shanghai Huyueming Scientific Instrument Co., Ltd., China) at 37°C. The OD value at 600 nm was measured and recorded every 2 h to plot the growth curve. Three biological replicates were performed for each group, and the bacterial growth curves were expressed as the mean value of turbidity, with absorbance corresponding to the time interval.

#### 2.6.4. Scanning electron microscopic

To confirm and validate the results observed with light microscopy, we examined the cellular structure of *S. aureus* by scanning electron microscopy according to a previously reported method (Bai et al., 2019) with slight modifications. *S. aureus* (10^8^ CFU/mL) was treated with the MIC concentration of UPPP at 37°C for 2 h. The bacteria were centrifuged at 6000 rpm for 10 min to precipitate and washed three times with PBS. The precipitated cells were fixed with 2.5% glutaraldehyde at 4°C for 24 h and then dehydrated with a series of different concentrations (15, 30, 45, 60, 75, 90 and 100%) of ethanol for 10 min. Finally, the dehydrated samples were coated with gold and examined by SEM (Tescan mira 2, Tescan, Europe, Czech Republic). A control experiment was performed without UPPP treatment.

### 2.7. Blueberry preservation

Blueberries of uniform size, color, and ripeness, free from mechanical damage, pests and diseases were selected and randomly divided into three groups of nine each. The blueberries were washed with sterile water, dried in aseptic operation at 25°C, immersed in different preservation solutions (0.02% potassium sorbate, UPPP at concentrations of 5 mg/mL and 25 mg/mL) for 15 min, and then, after the surface of the blueberries was dried, they were packed in special fruit packing boxes made of PET, and then stored in a refrigerator at 4°C for 7 days, and data such as weight and color change were measured every 24 h. Distilled water was used instead of the preservation solution as a control.

#### 2.7.1. Determination of decay rate

The decay rate of blueberries was detected using a modified version of a reported method (Wang et al., 2014). The fruit was divided into 4 classes according to the size of the fruit decay area (Wei and Zhao, 2008), and the calculation was done according to equation (1): the fruit softening and decay rate was divided into 4 classes according to the size of the fruit softening and decay area: class 0, no softening, decay, and no wrinkling of the epidermis; class 1, softening, decay, and wrinkling area <25% of the fruit area; class 2, softening, decay, and wrinkling area of 25 to 50% of the fruit area; class 3. Softening, decay, crumpling area is greater than 50% of the fruit area. Blueberries were observed for decay every 24 h. Three replicates were performed for each group. The decay rate was calculated by the following formula:


$$\mathrm{Decay rate}\;\left(\%\right)=\frac{\sum \mathrm{Decay level}\times \mathrm{Number of fruits}\;\mathrm{at}\;\mathrm{this level}}{\mathrm{Highest decay level}\times \mathrm{Total fruit quantity}}\times 100\%$$

#### 2.7.2. Determination of weight loss rate

The change in weight of blueberries for each day was measured using the weighing method. Three trials were repeated for each group of treatments. The rate of weight loss was expressed as a percentage of weight loss relative to the initial weight. The following formula was used to calculate the weight loss rate of blueberry:


$$\mathrm{Weight loss rate}\;\left(\%\right)=\frac{W_{0}-W_{n}}{W_{0}}\times 100\%$$

Where W_0_ and W_n_ are the weight of blueberry at 0 day and nth days, respectively.

#### 2.7.3. Determination of hardness

The hardness of blueberry was measured using a mass spectrometer (TA.TOUCH, Baosheng, Shanghai, China) with a probe diameter of 50 mm, a contact pressure of 20 gf, and a compression deformation rate of 40% using the TPA test method (pre-test speed of 2 mm/s, test speed of 1 mm/s, and post-test speed of 1 mm/s). Three trials were repeated for each group of treatments.

#### 2.7.4. Determination of color change

Blueberry is susceptible to browning during storage, and the color change can be reflected by the total color difference ΔE value. During storage, color parameters (L*, a*, b*) of blueberries from different treatment groups were measured daily using a colorimeter (CR-10 PLUS, Konica Minolta, Tokyo, Japan). Each set of experiments was repeated three times. The ΔE of blueberry was calculated as follows:


$$\Delta E=\sqrt{\left[{\left(\Delta L^{*}\right)}^{2}+{\left(\Delta a^{*}\right)}^{2}+{\left(\Delta b^{*}\right)}^{2}\right]}$$

Where ΔL^∗^, Δa^∗^, and Δb^∗^ are the change of blueberry at 0 day to 7 days. L^∗^ represents the black and white value, a^∗^ represents the red and green value, b^∗^ represents the yellow and blue value.

#### 2.7.5. Determination of soluble solids

Blueberries of different treatment groups were wrapped with gauze, squeezed out the juice and dripped in the center of the detection groove of the saccharimeter (PAL-1, ATAGO, Janpan), and left to stand for 1 min, then pressed the start button to start the detection, and the number shown on the display was the soluble solids of the blueberries. Each set of experiments was repeated three times.

#### 2.7.6. Determination of enzymatic activities of blueberries

The blueberries of different treatment groups were taken out from the storage environment, put into homogenization bags and added with the same quality of deionized water, and put into a beat homogenizer to homogenize at the speed of 10 for 90s to get the blueberry homogenate.

POD activity (U/g FW): Blueberries from day 0 and day 7 of storage were prepared as homogenates which were measured according to the steps described in the POD activity kit (Beijing Solepol Technology Co., Ltd., China) to calculate the POD activity of the blueberries. Three biological replicates were performed.

SOD activity (U/g FW): Homogenized blueberries from day 0 and day 7 of storage were measured as described in the SOD activity kit (Beijing Solepol Technology Co., Ltd., China) to calculate the SOD activity of blueberries. Three biological replicates were performed.

#### 2.7.7. Determination of total number of bacterial colonies

In each of the different treatment groups, 2.5 g of blueberries were placed in a sterile homogenization bag containing 22.5 mL of PBS buffer, and the homogenizer was agitated for 2 min to make a 1:10 homogenate of blueberries. Then, 1 mL of blueberry homogenate was injected into a test tube containing 9 mL of PBS buffer and shaken well to make a 1:100 sample homogenate, and so on to prepare a 10-fold series of diluted blueberry homogenate. A sterilized pipette was used to suck 1 mL of blueberry homogenate into the center of the colony test slice, and left to stand for 5 min, and the transparent side was upward after the Plate Count Agar (PCA) medium solidified, and then placed in a 37°C constant temperature incubator to observe the colonies and count them after 48 h of incubation (Zhu et al., 2021). Each set of experiments was repeated three times. The total number of colonies was calculated using the following formula:


$$\mathrm{Total number of colonies}\;\left(\mathrm{CFU}/\mathrm{mL}\right)=\frac{C\times D}{V}$$

C: number of colonies on the colony test piece (CFU); D: dilution multiple; V: the volume of blueberry homogenate used for testing (mL).

### 2.8. Statistical analysis

All experiments were performed three times using freshly prepared samples, and results were reported as the mean and standard deviation of these measurements. Statistical analysis was performed using Origin 2021, Graphpad Prism 8 and SPSS. To compare differences between multiple groups, analysis of variance (ANOVA) was performed on the data. *p* < 0.05 were considered statistically significant.

## 3. Results

### 3.1. Analysis of extraction and purification contents

The ultrasonic extraction yielded UPPP content of 70.05 ± 0.12 mg/g, and separation and purification with macroporous resin AB-8 resulted in UPPP purity of 87.56%. The sample was then freeze-dried, resulting in a brown powder.

### 3.2. Analysis of FT-IR spectroscopy

**Figure 2:**
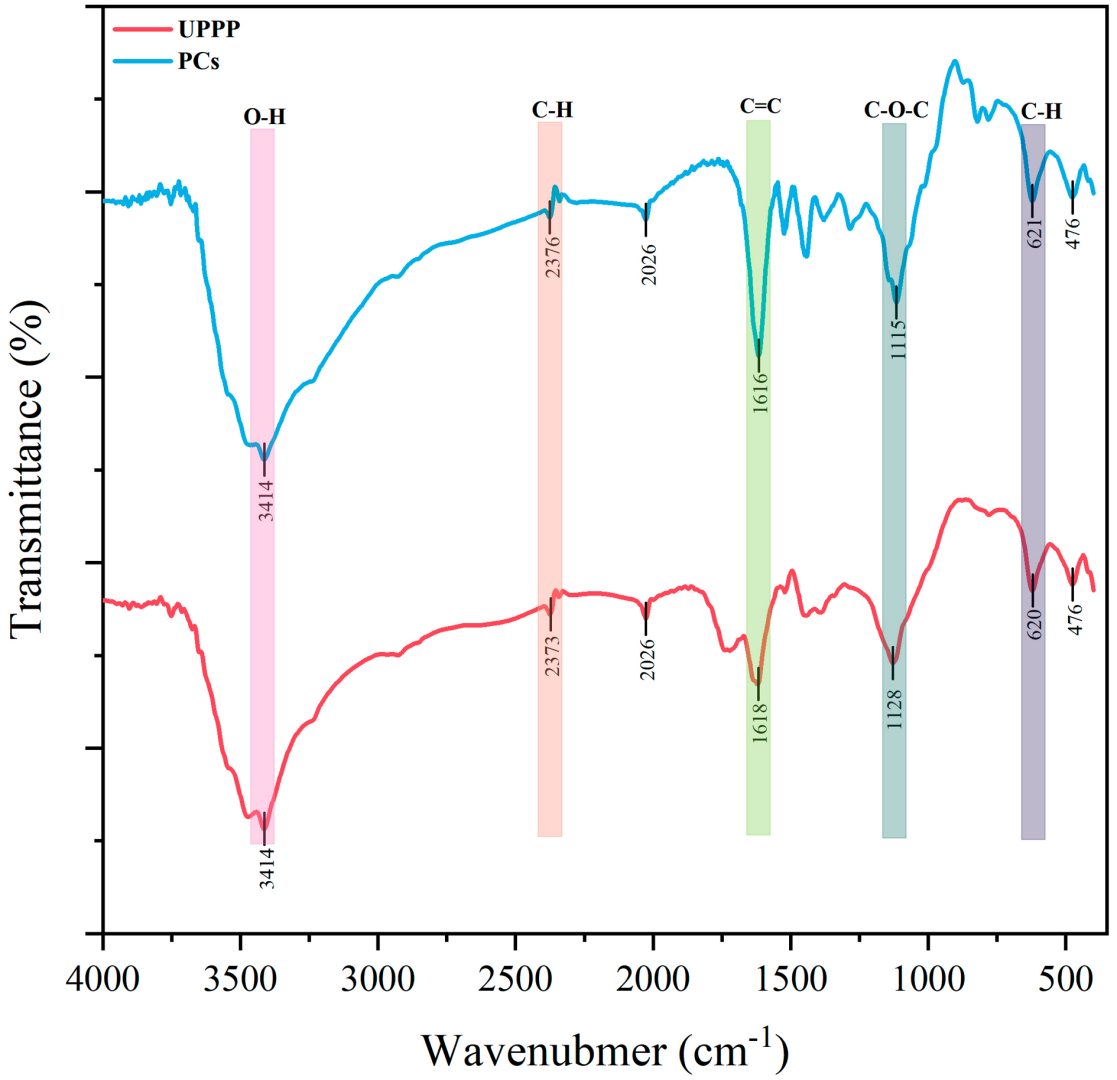
The results of FT-IR spectrograms of PCs standards and UPPP.

The IR spectra of UPPP and PCs standards are shown in Figure 2. From the Figure 2, it was found that the IR spectra of UPPP and PCs standards were very similar. UPPP had an IR absorption peak at 1522 cm^−1^, which indicated that the mass fraction of the structural unit procyanidin was higher than that of prodelphinidins in UPPP, and it was thus clear that the structural unit of UPPP was mainly procyanidin. The PCs dominated by prodelphinidins have an absorption peak near 730 cm^−1^, while the PCs dominated by procyanidin have an absorption peak near 770 cm^−1^ – 780 cm^−1^, which is attributed to their higher out-of-plane deformation vibration frequency than that of the PCs dominated by prodelphinidins, and the infrared absorption peak in UPPP at 779 cm^−1^ also confirms that the main structural unit of UPPP is procyanidin (Foo, 1981).

### 3.3. Analysis of physicochemical properties of UPPP

Under acidic conditions, the color of the solution of UPPP changed to red after shaking following the addition of NH₄Fe(SO₄)₂ or vanillin (Table 1). The above two identification reactions were consistent with the reaction characteristics of PCs, indicating that UPPP did contain PCs.

**Table 1:** PCs identification reaction of UPPP.

Testing indicators	UPPP
NH₄Fe(SO₄)₂ experiment	+
Vanillin experiment	+

+, Positive.

### 3.4. Antibacterial activity

#### 3.4.1. Bacterial inhibition zone

**Figure 3:**
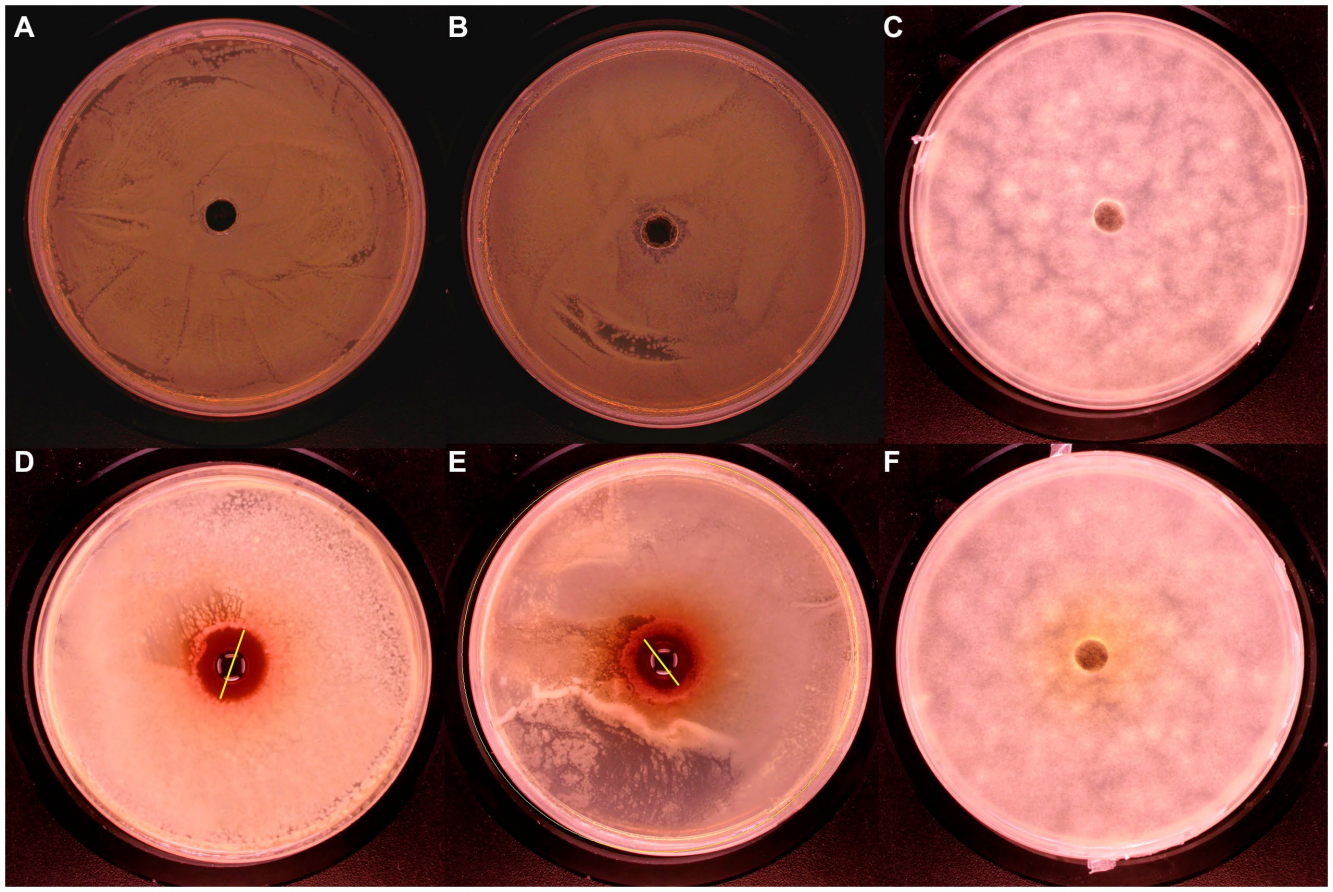
Bacteriostatic effect of UPPP on *S. aureus*, *E. coli* and *A. niger*. [**(A–C)** Blank controls for *S. aureus*, *E. coli* and *A. niger*, respectively; **(D)** *S. aureus*; **(E)** *E. coli*; **(F)** *A. niger*].

The inhibition ability of UPPP was determined by punching method, and it was shown in Figure 3 that UPPP has growth inhibition effect on *S. aureus*, and *E. coli*, but not on *A. niger*. The results of the bacterial zone of inhibition diameters were shown in Table 2, where the zone of inhibition diameters for *S. aureus* were 20.41 ± 1.34 mm and for *E. coli* were 12.97 ± 0.05 mm. The experiments proved that UPPP has good bacterial inhibition ability.

**Table 2:** Inhibitory effect of UPPP on three experimental bacteria.

Group	Diameter of bacterial inhibition zone of different system categories (mm)		
	*S. aureus*	*E. coli*	*A. niger*
Control	–	–	–
UPPP	20.41 ± 1.34^a^	12.97 ± 0.05^b^	–

“–” It means no bacteriostatic effect. Each value represents mean ± SD of triplicates. Different lowercase letters indicate significant differences for *p* < 0.05.

#### 3.4.2. Minimum inhibitory concentration

The MIC of UPPP was determined as shown in Table 3. The MIC of UPPP was 1.563 mg/mL for *S. aureus*, 3.125 mg/mL for *E. coli*, in contrast, at a mass concentration of 25 mg/mL of UPPP, there was no inhibitory effect on *A. niger*. It can be seen that UPPP has a good inhibitory effect on bacteria, especially *S. aureus*.

**Table 3:** Inhibitory effect of UPPP on three experimental bacteria.

Group	Mass concentration of UPPP (mg/mL)												MIC
	25	12.5	6.25	3.125	1.563	0.781	0.39	0.195	0.098	0.049	0.024	0.012	
*S. aureus*	−	−	−	−	−	+	+	+	+	+	+	+	1.563
*E. coli*	−	−	−	−	+	+	+	+	+	+	+	+	3.125
*A. niger*	+	+	+	+	+	+	+	+	+	+	+	+	/

“+” Indicates microbial growth; “–” indicates no microbial growth; “/” indicates that there was no MIC in the above concentration range.

#### 3.4.3. Growth curve

**Figure 4:**
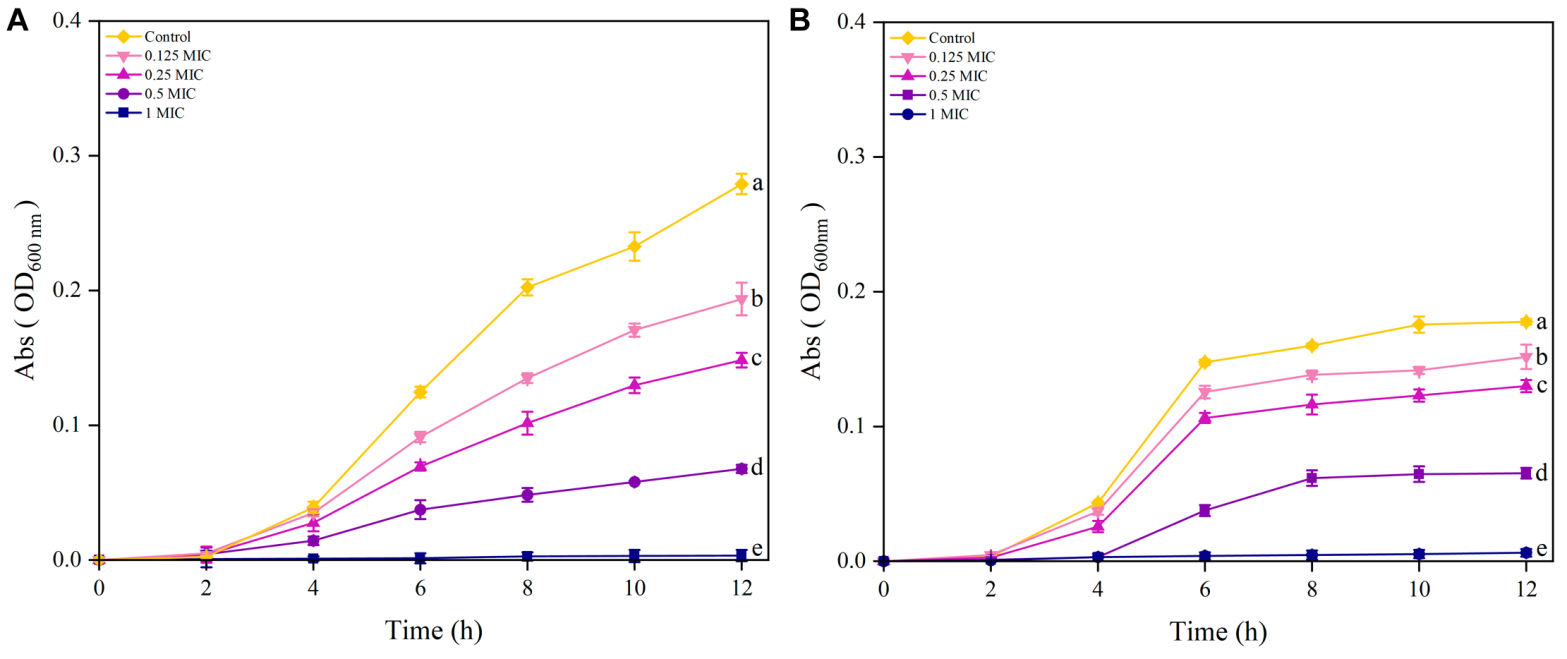
Growth curves of *S. aureus* **(A)** and *E. coli* **(B)** after treatment with different concentrations of UPPP. (Different lowercase letters indicate significant differences for *p* < 0.05).

As shown in Figure 4A the results showed that *S. aureus* in the control group reached the exponential growth phase after 4 h and did not enter the stable phase after 12 h. After treatment with 0.125 MIC, 0.25 MIC and 0.5 MIC of UPPP, *S. aureus* entered the exponential growth phase after 4 h and none of them reached the stable phase after 12 h. The absorbance of 1 MIC did not change in 12 h, indicating that *S. aureus* The absorbance of 1 MIC did not change at 12 h, indicating that *S. aureus* was very sensitive to 1 MIC concentration; different concentrations of UPPP had inhibitory effect on *S. aureus* at 12 h.

The results in Figure 4B indicated that the growth of *E. coli* treated with Control, 0.25 MIC, and 0.125 MIC entered the exponential growth phase after 4 h of incubation and reached the stationary phase at 6 h. The growth curve of *E. coli* treated with 0.5 MIC of UPPP lagged behind the control group by 2 h and entered the stabilization period at 8 h. Furthermore, 1 MIC completely inhibited the growth of *E. coli* within 12 h.

#### 3.4.4. Analysis of SEM

**Figure 5:**
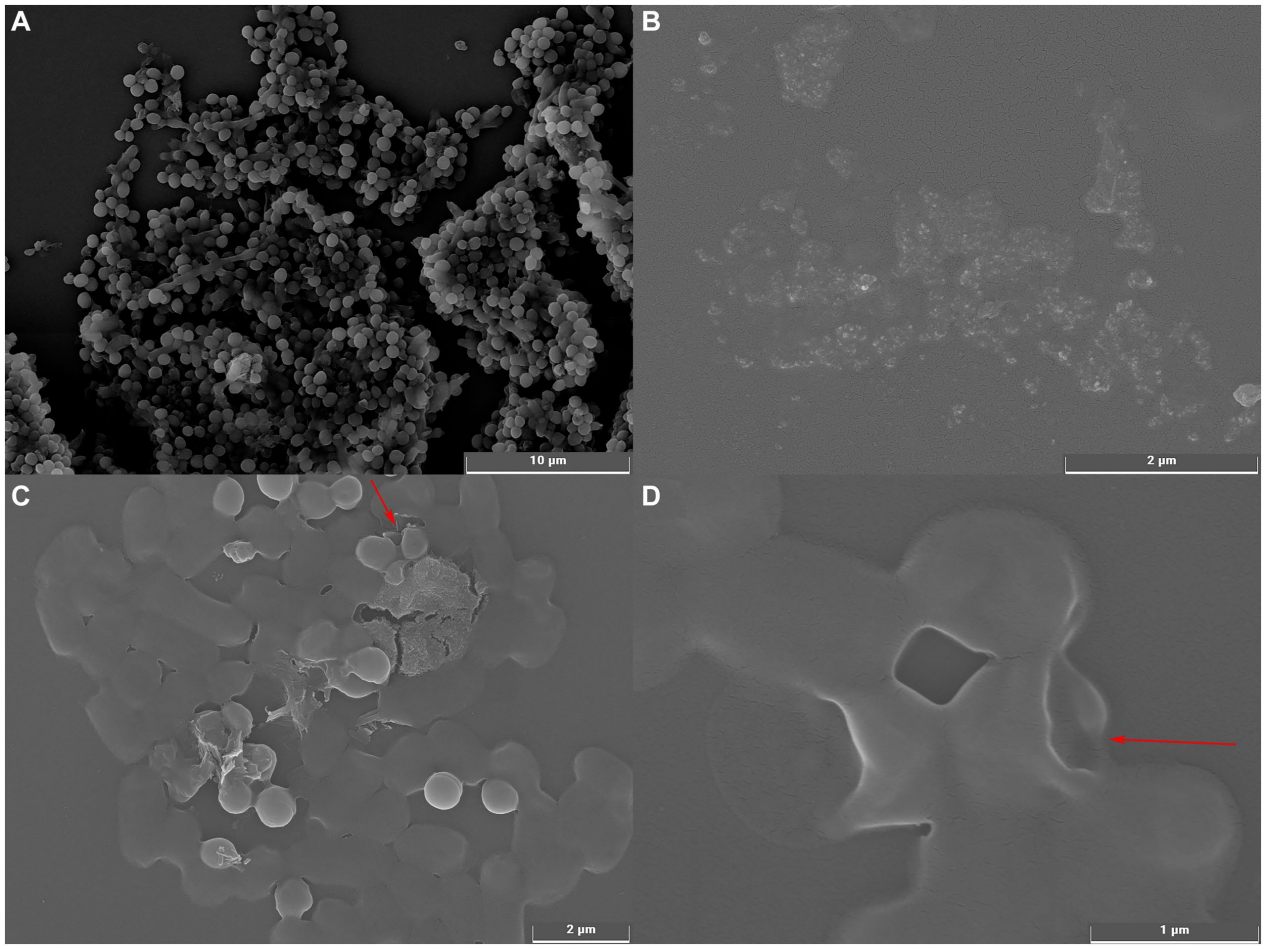
Scanning electron microscopy of *S. aureus* before and after UPPP treatment. [**(A)** *S. aureus* before UPPP treatment; **(B)** UPPP; **(C,D)** *S. aureus* after UPPP treatment].

The effect of UPPP on the morphology of *S. aureus* bacterial cells was observed by using scanning electron microscope and the results were shown in Figure 5. Figure 5A showed the cell morphology of *S. aureus* cultured under normal conditions with complete cellular morphology, the bacterial cell surface was complete and smooth overall presenting round spherical cells, the bacterial body was full and tightly structured. Figures 5C,D showed that after *S. aureus* was treated with MIC concentration of UPPP, the bacterial surface showed varying degrees of deformation, unclear outline, surface collapse, cell membrane depression, breakage, and the appearance of aberrant cells, which indicated that the cell wall and cell membrane structure of *S. aureus* were damaged by UPPP, and the incompleteness of the cell wall and cell membrane structure led to the leakage of cellular contents, which caused the bacterium to lose its integrity, resulting in bacterial suppressive effect.

### 3.5. Blueberry preservation

#### 3.5.1. Analysis of decay rate

**Figure 6:**
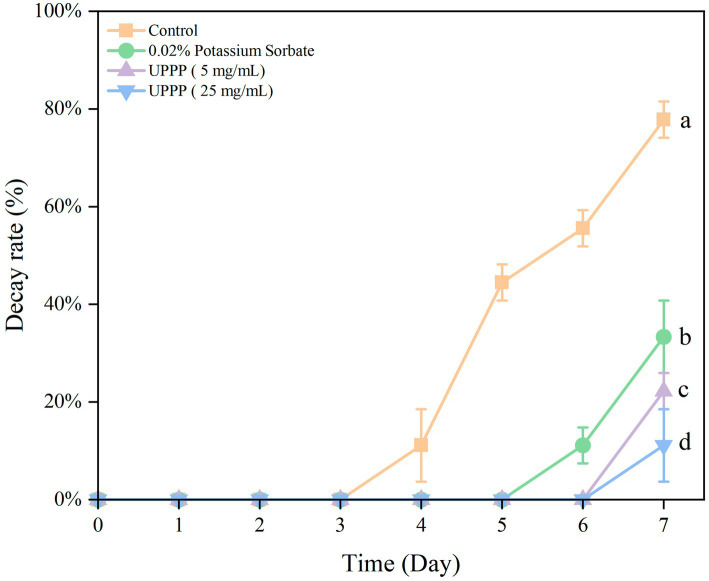
The effect of UPPP on decay rate of blueberry stored for 7 days. (Different lowercase letters indicate significant differences for *p* < 0.05).

The results of the decay rate of blueberry stored for 7 days are shown in Figure 6, with the extension of the storage time of blueberry, the number of rotten blueberries showed an upward trend. From the beginning of storage to 7 days of storage, the decay rate of the Control group was significantly higher than that of the treatment group (*p* < 0.05). In conclusion, the treatment group can reduce the decay rate of blueberries during storage, and 25 mg/mL UPPP has the best effect on delaying the decay of blueberries.

#### 3.5.2. Analysis of weight loss rate

**Figure 7:**
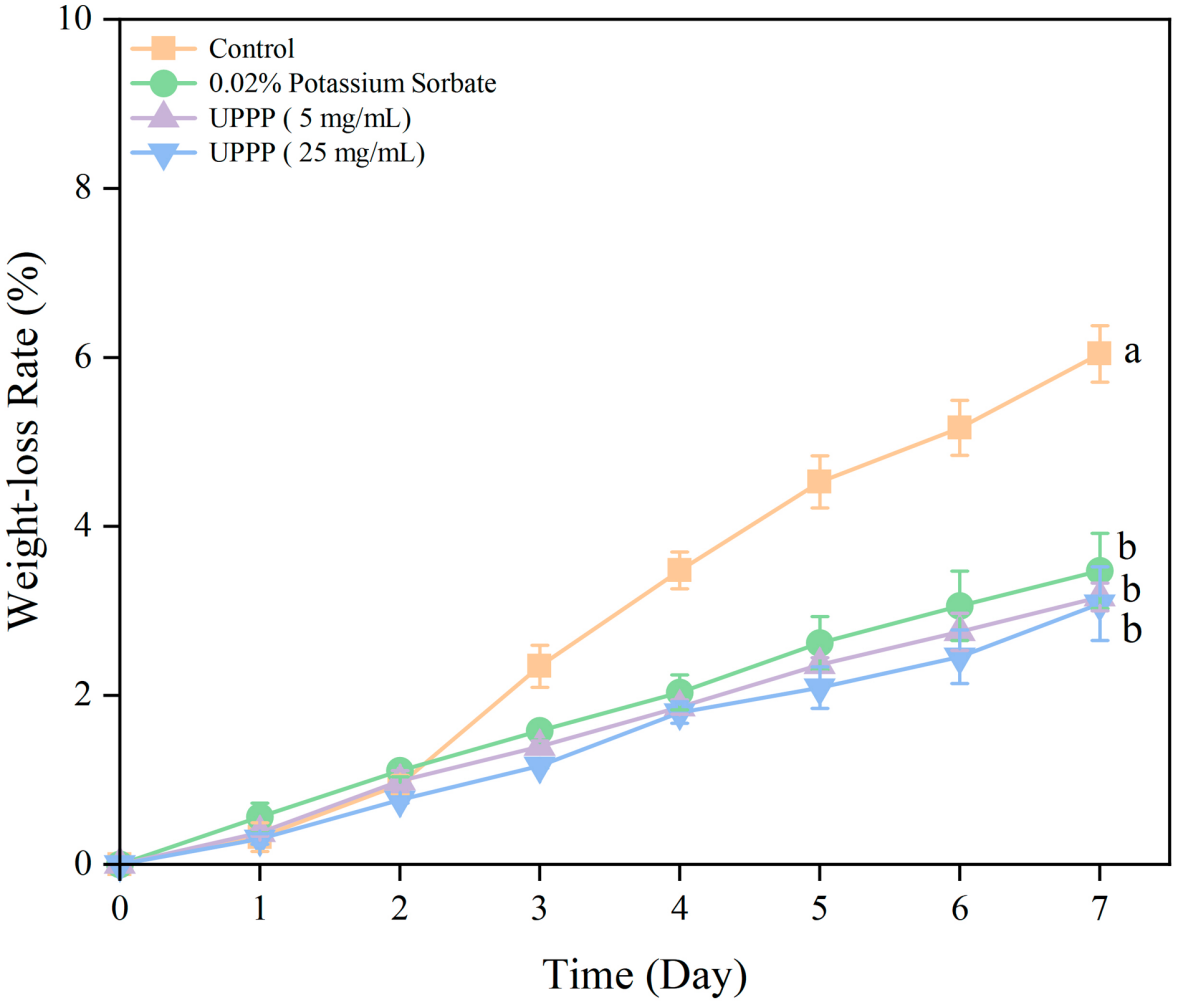
The effect of UPPP on weight loss rate of blueberry stored for 7 days. (Different lowercase letters indicate significant differences for *p* < 0.05).

The results of the 7-day experiment on weight loss rate of UPPP-treated blueberry is shown in Figure 7 where the weight loss rate of blueberry gradually increased in both treatment and Control groups. The treatment of blueberry with 0.02% potassium sorbate and UPPP at concentrations of 5 mg/mL and 25 mg/mL after 7 days of storage showed a significant difference (*p* < 0.05) in the weight loss rate when compared with the Control group, proving that the UPPP treatment was effective in reducing the weight loss of blueberry, with the concentration of 25 mg/mL resulting in the lowest rate of weight loss of blueberry.

#### 3.5.3. Analysis of hardness

**Figure 8:**
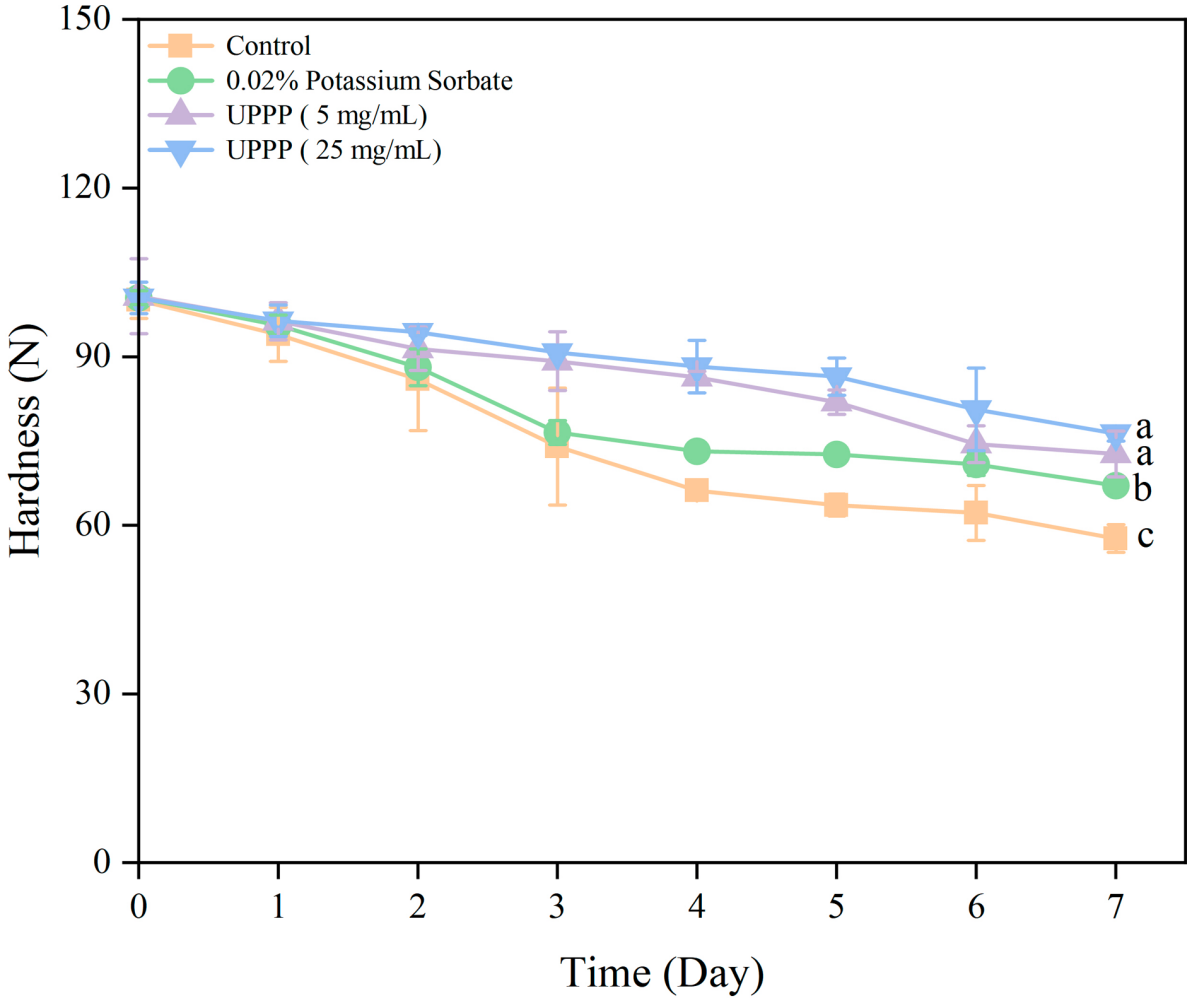
The effect of UPPP on hardness of blueberry stored for 7 days. (Different lowercase letters indicate significant differences for *p* < 0.05).

The 7-day experimental results of hardness of UPPP-treated blueberry is shown in Figure 8. The hardness of blueberry in both the treated and control groups gradually decreased, and compared with the initial value, the hardness of the control group decreased by 42.36% after 7 days of storage. The treatment groups were able to slow down the decline of blueberry hardness (*p* < 0.05), and the UPPP treatment group with a concentration of 25 mg/mL had the least change in hardness before and after storage, which was the most effective in maintaining the hardness of blueberry. The hardness of blueberry was maintained optimum.

#### 3.5.4. Analysis of color change

**Figure 9:**
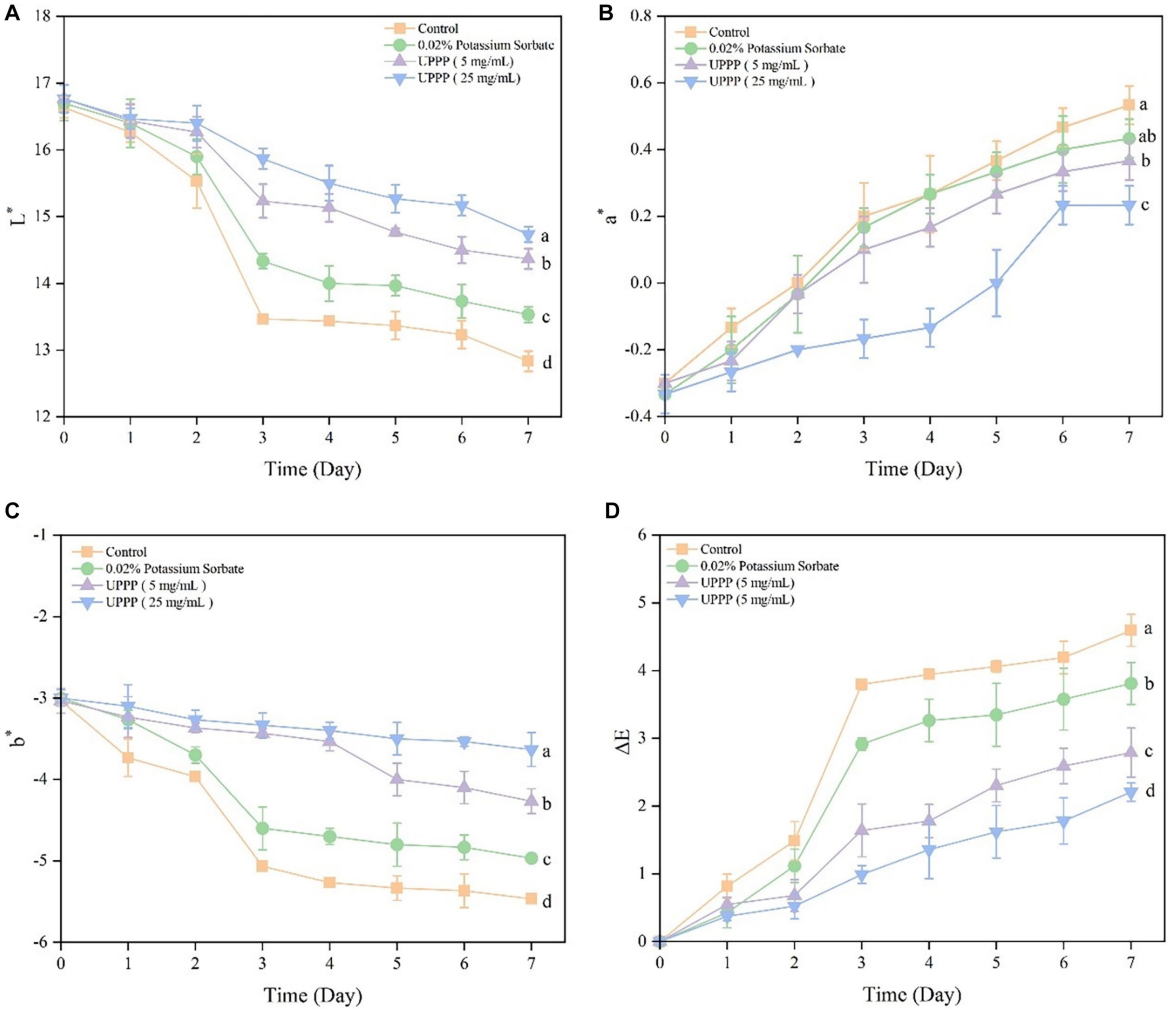
The effect of UPPP on color change of blueberry stored for 7 days. **(A)** represents the black and white value, **(B)** represents the red and green value, **(C)** represents the yellow and blue value, **(D)** represents total color difference (different lowercase letters indicate significant differences for *p* < 0.05).

The color change of UPPP-treated blueberry was assessed by measuring the L^*^ value (brightness), a^*^ value (which indicates red-green, with positive values indicating red, and larger values representing a darker red color of the samples), and b^*^ value (which indicates a yellowish-blue color, with negative values indicating blue, and smaller values representing a darker blue color). The experimental results are shown in Figure 9. During storage, the L^*^ and b^*^ values of the blueberry and Control groups treated with 0.02% potassium sorbate, UPPP at concentrations of 5 mg/mL and 25 mg/mL, decreased, and the a^*^ values increased, reflecting a decrease in brightness and an increase in surface color saturation. ∆E could reflect the overall change in color of blueberry during storage. There was a general trend of increasing ∆E during storage, which was consistent with the normal metabolism of blueberries. Compared with day 0, the ∆E of the control, 0.02% potassium sorbate-treated, 5 mg/mL UPPP-treated, and 25 mg/mL UPPP-treated groups were 4.59 ± 0.24, 3.81 ± 0.31, 2.79 ± 0.36, and 2.21 ± 0.13, which were significantly different (*p <* 0.05) in all the blueberry-treated groups when compared to the control group, and the UPPP treatment groups were all <0.02% potassium sorbate, indicating that the UPPP treatment was able to maintain the color change of blueberries.

#### 3.5.5. Analysis of soluble solids

**Figure 10:**
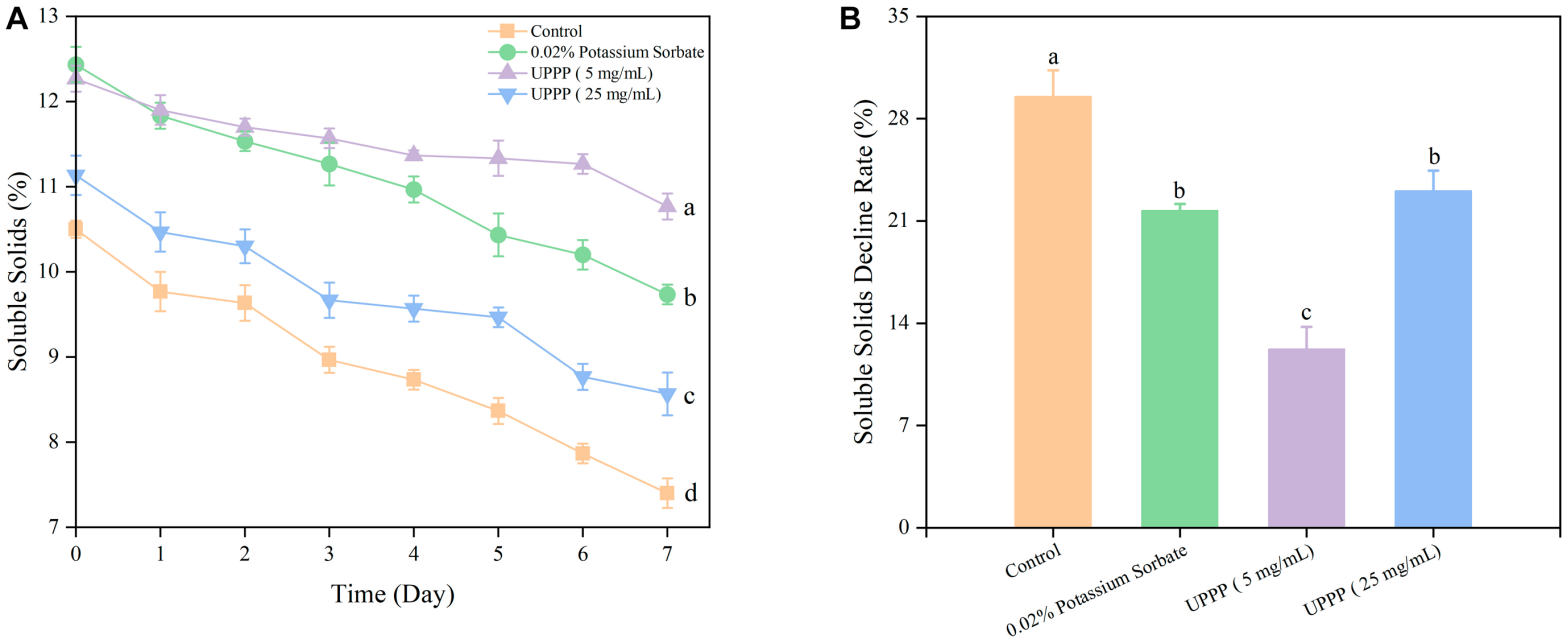
The effect of UPPP on soluble solids of blueberry stored for 7  days. **(A)** represents soluble solids, **(B)** represents soluble solids decline rate (Different lowercase letters indicate significant differences for *p* < 0.05).

Soluble solids is an important indicator of fruit ripening and senescence, and the results of 7-day experiments of soluble solids and soluble solids decline rate of UPPP-treated blueberries were shown in Figure 10, with the prolongation of the storage time, soluble solids were consumed as respiratory substrates, and the soluble solids were all in a decreasing trend. After 7 days of storage, treatment of blueberry with 0.02% potassium sorbate and UPPP at concentrations of 5 mg/mL and 25 mg/mL showed a significant (*p* < 0.05) difference in the rate of decrease of soluble solids when compared with the Control group, implying that different concentrations of UPPP had an effect on the soluble solids of blueberry. Among them, UPPP at a concentration of 5 mg/mL was effective in reducing the consumption of soluble solids.

#### 3.5.6. Enzymatic activities of blueberries

**Figure 11:**
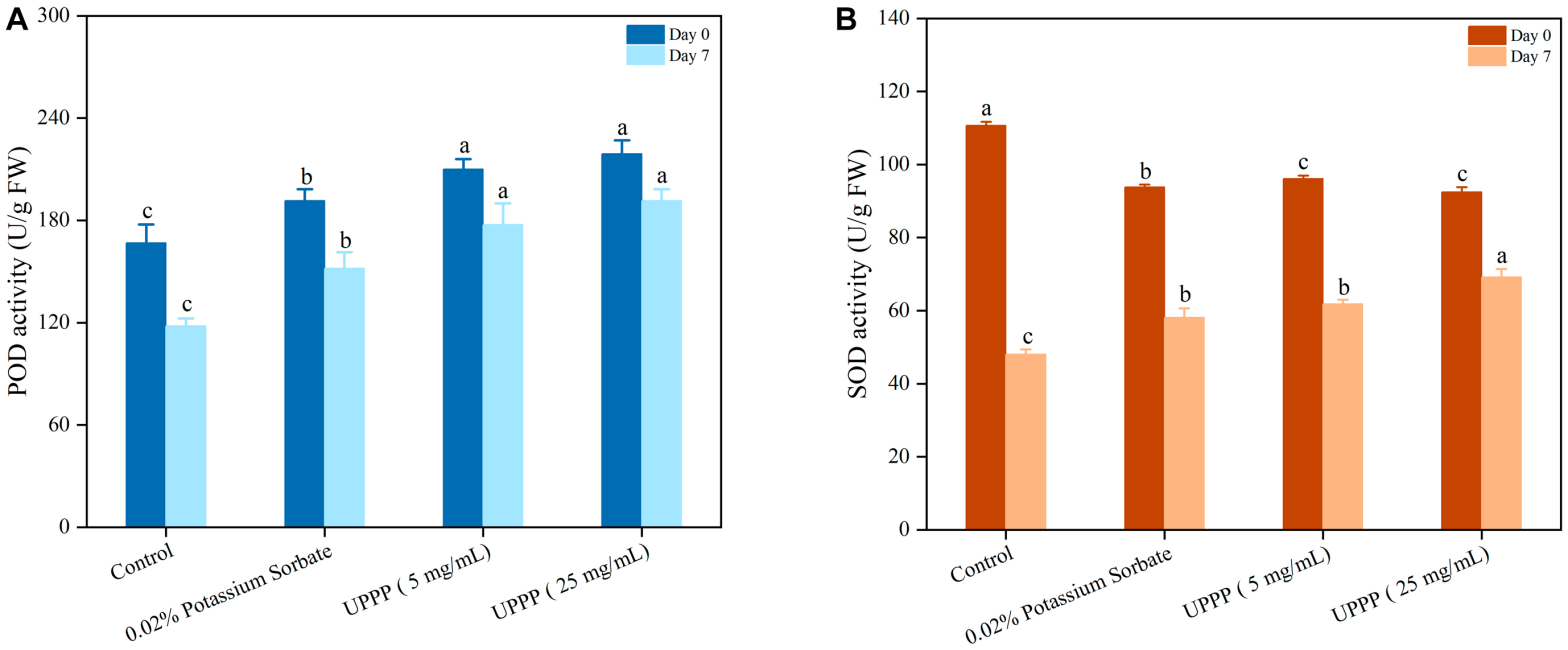
The effect of UPPP on POD activity **(A)** and SOD activity **(B)** of blueberry stored for 7 days (different lowercase letters indicate significant differences for *p* < 0.05).

Figure 11A showed higher POD activity on day 0. After 7 days of blueberry storage at 4°C, the POD activity of the control group was 117.98 ± 4.54 U/g FW. It was 22, 34 and 38% lower than the 0.02% potassium sorbate, 5 mg/mL and 25 mg/mL UPPP treatment groups, respectively, indicating that the treatment groups had higher POD activity than the control group after 7 days of blueberry storage, especially the 25 mg/mL UPPP treatment group. There was a significant difference (*p* < 0.05) in POD activity between the treated and control groups after 7 days of storage, suggesting that UPPP was able to better limit the membrane peroxidation process, thus maintaining the integrity of blueberry cells.

As shown in Figure 11B, the content of SOD determined the rate of oxygen radical production by fruit and vegetable cells during storage. At the beginning of the storage period, mechanical damage resulted in elevated SOD enzyme activity, but after 7 days of storage at 4°C, SOD activity was significantly higher in the treatment group than in the control group (*p* < 0.05). At the end of the storage period, the SOD activities of the control, 0.02% potassium sorbate-treated, 5 mg/mL UPPP-treated, and 25 mg/mL UPPP-treated groups were 48.04 ± 1.32 U/g FW, 58.13 ± 2.50 U/g FW, 61.83 ± 1.17 U/g FW, and 69.20 ± 2.20 U/g FW, respectively, indicating that 25 mg/mL UPPP was important in maintaining the activity of reactive oxygen scavenging enzymes, preventing the accumulation of reactive oxygen species and the occurrence of membrane lipid peroxidation, delaying senescence and inhibiting fruit softening in blueberry. In conclusion, UPPP inhibited browning of blueberry by inducing POD activity and SOD activity to alleviate peroxidation.

#### 3.5.7. Analysis of total number of bacterial colonies

**Figure 12:**
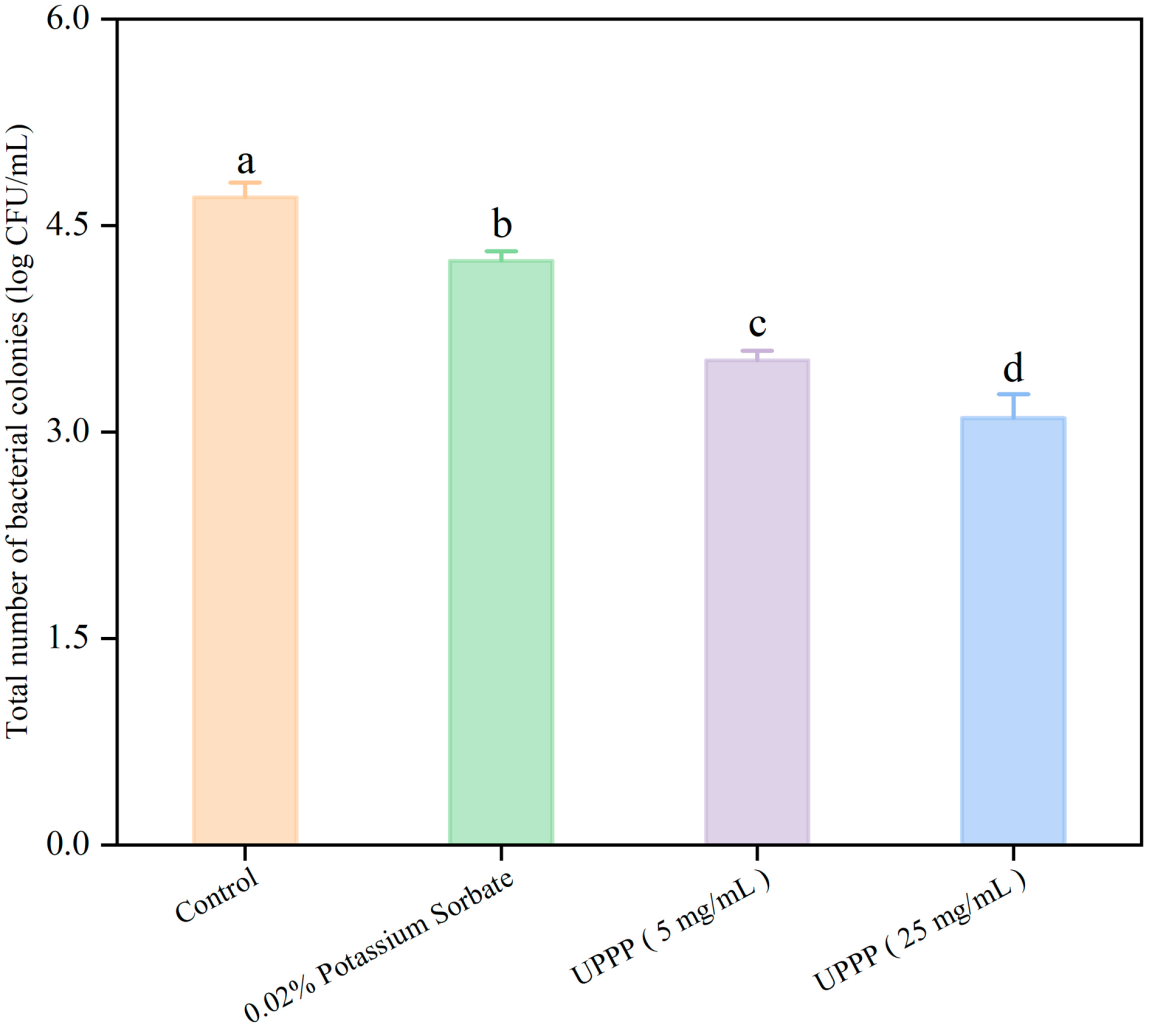
The effect of UPPP on total number of bacterial colonies of blueberry stored for 7 days (different lowercase letters indicate significant differences for *p* < 0.05).

As shown in Figure 12, the number of colonies of blueberries was assessed after different treatments over a period of 7 days. Compared with the control group (4.70 ± 0.11 log CFU/mL), the total number of colonies in the treatment group was lower. Specifically, the total number of blueberry colonies treated with 0.02% potassium sorbate was 4.24 ± 0.07 log CFU/mL. The total number of blueberry colonies treated with 5 mg/mL and 25 mg/mL UPPP was 3.52 ± 0.07 log CFU/mL and 3.10 ± 0.17 log CFU/mL. These differences between the treated group and the control group were statistically significant (*p* < 0.05). By comparing the total number of colonies of blueberries treated with different concentrations of UPPP and 0.02% potassium sorbate, it was found that UPPP treatment was the most effective, especially with a concentration of 25 mg/mL. The results showed that the different concentrations of UPPP had strong antibacterial activity, which could significantly inhibit the total number of colonies of blueberries during the storage process, and thus reduced the microbial infestation of blueberries and the rate of rotting.

## 4. Discussion

Although PCs have been shown to act as natural food preservatives (Jeong et al., 2015), the application of UPPP in natural food preservatives has not yet been investigated (Jiang et al., 2022). Fresh fruits and vegetables are very susceptible to microbial attack (e.g., foodborne pathogens) during preservation, leading to rapid spoilage and shortened shelf-life, so there is a great need for natural food preservatives with superior antimicrobial capacity, and according to existing reports, PCs have strong antimicrobial activity against *S. aureus* (Jekabsone et al., 2019), which is in agreement with the present experimental results of the study. Other authors have reported similar antimicrobial effects when testing PCs; for example, Garzón et al. (2020) observed that *S. aureus* was susceptible to procyanidins isolated from Vaccinium meridionale Swartz pomace (Garzón et al., 2020), and Li et al. (2017) reported the inhibitory effect of Larch bark PCs on *S. aureus* growth (Li et al., 2017).

In the IR spectrogram of UPPP (Figure 2), 3,414 cm^−1^ is the absorption peak of -OH stretching vibration of polyphenols with a broader peak shape, which is attributed to the effect of hydrogen bond formation between more phenolic hydroxyl groups on the benzene ring of PCs; 2,373 cm^−1^ is the asymmetric stretching vibration of polyphenol C-H bond (İsmet et al., 2010); the weaker stretching vibration observed at 1618 cm^−1^ is the C=C backbone stretching vibration absorption peak on the benzene ring of polyphenolic molecules (Mahendran et al., 2021); and 1,128 cm^−1^ is the anti-symmetric stretching vibration absorption peak of the C-O-C ether bond on the heterocyclic ring (Cui et al., 2012). Three hydroxyl groups are present in the B ring of PCs with prodelphinidins as the structural unit, and two absorption peaks are present at 1540 cm^−1^ – 1520 cm^−1^, while the B ring of PCs with procyanidin as the structural unit is the presence of two hydroxyl groups, and only one absorption peak is present at 1540 cm^−1^ – 1535 cm^−1^ (Li et al., 2005). Therefore, the UPPP extracted in this experiment belongs to PCs and contains phenolic hydroxyl structure. It has been shown that the molecular structure of PCs contains a large number of phenolic hydroxyl groups, which can damage cell membranes and increase the permeability of bacterial membranes, leading to intracellular soluble efflux and reduced indirect metabolism or metabolic disorders due to the loss of ATP and other intracellular metabolites (Di et al., 2007; Dinesh et al., 2012).

In the study, it was observed that only *S. aureus* in the 1 MIC treatment group reached a stable phase within 12 h. This result could be explained by an insufficient dosage of UPPP. When the concentration of antibacterial agents was too low, it might not have fully inhibited the growth and reproduction of *S. aureus*, leading to ongoing proliferation and failure to reach stability. The number of *S. aureus* could have also impacted its growth and reproduction, further delaying the attainment of a steady state. Environmental conditions could have also affected the physiological and metabolic adjustments of *S. aureus*, making it challenging to quickly reach a stable phase. Antibacterial agents affect the morphology of bacterial cells in various ways, such as separation of the cytoplasmic membrane from the cell wall, leakage of cytoplasmic contents, cell lysis and cell deformation (Teng et al., 2014; Shen et al., 2015). In this study, it was found that UPPP was able to disrupt the morphology of *S. aureus*. Therefore, it was hypothesized that the bacterial ultrastructural changes might be due to the action of UPPP leading to cell lysis and a dramatic effect on the cell membrane, resulting in a massive leakage of cellular components. Other studies have shown that PCs have anti-adhesion effects on specific bacteria, achieved by decreasing the levels of colonies in areas such as sugar metabolism and the expression of genes associated with bacterial adhesion (Lin et al., 2011). However, more studies are needed to elucidate the mechanism of the antibacterial effects of PCs.

The appearance of blueberry proved to be severely affected by browning and dehydration after receiving treatment in the blank group. However, the UPPP-treated blueberry kept their appearance intact, and then the quality evaluation of the blueberry was derived from a number of indicators. First, comparing the weight loss of control and experimental groups stored at 4°C for 7 days, it was found that the weight loss rate of the experimental group was significantly lower than that of the control group, suggesting that the UPPP treatment was effective in reducing the weight loss of blueberry; This was probably due to the fact that when UPPP was applied to the surface of blueberries, the hydrophobic groups in UPPP exerted a strong force on the water molecules and had a strong water barrier effect, reducing the evaporation of water through the pores of the blueberry epidermis to affect the permeability of the blueberries to CO_2_, O_2_, and water vapour, thus saturating the internal pressure and thus reducing the rate of weight loss (Nor and Ding, 2020). As a result, the weight loss of blueberries in the UPPP group was lower than that of the control group. Hardness is also an important indicator of fruit ripeness and quality evaluation, as degradation of cell wall components during storage brings about loss of hardness and even microbial invasion. Exogenous polyphenols can bind to pectin in the cell walls of fruits and vegetables and inhibit softening of fruits and vegetables. PCs can bind to pectin through hydrogen bonding, hydrophobic bonding, ionic bonding and covalent bonding. In addition, PCs can also reduce pectinase activity, and thus have a certain effect on the cell wall of fruits and vegetables (Ren et al., 2019). Some researchers have used 1% PCs to treat bananas and found that PCs-treated fruits exhibited better hardness compared to control banana fruits (Chen et al., 2022). This was consistent with the results of the present experimental study.

The external color of fresh fruits is one of the most important factors in determining consumer acceptance of a product (Del-Valle et al., 2004). The L^*^ and b^*^ values of blueberries gradually decreased during storage, while the a^*^ values gradually increased, reflecting a decrease in brightness and an increase in surface color saturation. Blueberries in the UPPP treatment group showed minimal color changes, which was effective in maintaining the color of blueberries. POD and SOD are important active enzymes present in plants, POD can act synergistically with catalase to decompose H_2_O_2_ into H_2_O and O_2_ in plants, and SOD can catalyze the conversion of superoxide anion into H_2_O_2_ and O_2_, both of which can reduce cellular damage caused by reactive oxygen radicals, and the activities of these enzymes are also closely correlated with the ripening degree of blueberry fruits (Liang et al., 2022). The decreased POD activity and SOD activity of blueberries after 7 days of storage was mainly due to the senescence of blueberries, which produced excess free radicals to inhibit the enzyme activities (Chen et al., 2013). The results showed that POD activity and SOD activity were higher after UPPP treatment than in the control group, indicating that UPPP could maintain POD and SOD activities and protect cells from damage. Similar results were observed after coating lychee pericarp with PCs (Shen et al., 2023). Importantly, in the UPPP treatment group, 25 mg/mL of UPPP showed the best preservation performance in blueberry, with the smallest changes in the indexes of decay rate, hardness, and color change, the highest POD and SOD activities, and the lowest total number of colonies, suggesting that the optimal concentration of UPPP for use in the application of blueberry preservation should be 25 mg/mL. In conclusion, UPPP can be used as a natural food preservative to extend the shelf life of small fruit. It provides a research basis and direction for the development and utilization of UPPP in the pharmaceutical, food and health care industries, and improves the added value of UPPP resources.

The results of this study provided valuable insights into the potential applications of ume-derived preservatives in food preservation, but several aspects need to be addressed in order to utilize them on a wider scale. One of the primary challenges associated with the widespread use of ume-derived preservatives is the availability and scalability of production. A mass production method should be developed to meet the growing demand. Additionally, the sourcing of high-quality ume plums, as well as the extraction and purification processes, should be optimized to ensure consistent and efficient production. Each fruit and vegetable has unique properties that may affect the effectiveness of preservatives (Teshome et al., 2022). Further research should focus on evaluating the stability and compatibility of UPPPs with different food matrices by investigating potential interactions between UPPPs and various food ingredients. In addition, long-term stability studies and comprehensive toxicological evaluations are needed to ensure that UPPP is suitable for commercial use. To address these challenges, future research should focus on establishing collaborations between academic institutions, food industry stakeholders, and government bodies. Such collaborations can facilitate the development of standardized production processes, formulation techniques, and quality control measures for UPPP-based preservatives. Additionally, implementing good agricultural practices for ume plum cultivation can help ensure a sustainable and consistent supply of raw materials. In conclusion, the wider application of ume-derived preservatives requires addressing challenges associated with production scalability, compatibility with various food matrices, and long-term stability and safety. Through collaborative efforts and a multidisciplinary approach, these challenges can be overcome, opening avenues for the utilization of UPPP-based preservatives in the food industry.

However, this study is still deficient in that the inhibition of *Salmonella typhimurium* and *Listeria monocytogenes* by UPPP was not evident in preliminary experiments and the ability of this preservative to inhibit these two foodborne pathogens was not analyzed. In addition, monthly variations may affect the concentration of PCs in ume, and side effects in humans have not been studied at the doses used or at high doses. Subsequent experiments will look more specifically at the composition and content of the experimental PCs, using HPLC or LC–MS instrumentation to quantify and characterize the purified extracts. In recent years, with the intensive research and application development of umeboshi, related products have been released. Therefore, the development of new foods, health foods and drugs from ume is promising.

## 5. Conclusion

In this paper, we investigated the antibacterial properties of UPPP and its preservation effect on blueberry. UPPP showed the best antibacterial effect against *S. aureus* (20.41 ± 1.34 mm), but poorer antibacterial effect against *E. coli* (12.97 ± 0.05 mm). The physiological and biochemical indexes of blueberry stored at 4°C for 7 d after treatment were determined. The results showed that the decay rate, weight loss rate, hardness, color change, soluble solids decline rate and total colony number of blueberry in the UPPP-treated group were lower than those in the blank control group and the 0.02% potassium sorbate group; and the POD activity and SOD activity were higher than those in the blank control group and the 0.02% potassium sorbate group. In conclusion, UPPP is a natural food preservative, which can significantly increase the shelf life of blueberry, providing a scientific basis for the application of UPPP in the preservation of blueberry.

## Data availability statement

The original contributions presented in the study are included in the article/supplementary material, further inquiries can be directed to the corresponding author.

## Author contributions

LL: Conceptualization, Data curation, Formal analysis, Funding acquisition, Investigation, Methodology, Project administration, Resources, Software, Supervision, Validation, Visualization, Writing – original draft, Writing – review & editing. HQ: Writing – review & editing. YuL: Writing – review & editing. YiL: Writing – review & editing. LW: Writing – review & editing. WZ: Writing – review & editing. FM: Writing – review & editing.
